# Determinants of successful clinical networks: the conceptual framework and study protocol

**DOI:** 10.1186/1748-5908-7-16

**Published:** 2012-03-13

**Authors:** Mary Haines, Bernadette Brown, Jonathan Craig, Catherine D'Este, Elizabeth Elliott, Emily Klineberg, Elizabeth McInnes, Sandy Middleton, Christine Paul, Sally Redman, Elizabeth M Yano

**Affiliations:** 1 Sax Institute, Haymarket, Australia; 2 School of Public Health, University of Sydney, Camperdown, Australia; 3 Centre for Clinical Epidemiology and Biostatistics, University of Newcastle, Newcastle, Australia; 4 The University of Sydney Clinical School, The Children's Hospital at Westmead, Westmead, Australia; 5 Nursing Research Institute - St. Vincents & Mater Health Sydney and Australian Catholic University, National Centre for Clinical Outcomes Research (NaCCOR), Darlinghurst, Australia; 6 School of Medicine and Public Health, University of Newcastle, Newcastle, Australia; 7 VA Greater Los Angeles HSR&D Centre of Excellence, Sepulveda, CA, USA

## Abstract

**Background:**

Clinical networks are increasingly being viewed as an important strategy for increasing evidence-based practice and improving models of care, but success is variable and characteristics of networks with high impact are uncertain. This study takes advantage of the variability in the functioning and outcomes of networks supported by the Australian New South Wales (NSW) Agency for Clinical Innovation's non-mandatory model of clinical networks to investigate the factors that contribute to the success of clinical networks.

**Methods/Design:**

The objective of this retrospective study is to examine the association between external support, organisational and program factors, and indicators of success among 19 clinical networks over a three-year period (2006-2008). The outcomes (health impact, system impact, programs implemented, engagement, user perception, and financial leverage) and explanatory factors will be collected using a web-based survey, interviews, and record review. An independent expert panel will provide judgements about the impact or extent of each network's initiatives on health and system impacts. The ratings of the expert panel will be the outcome used in multivariable analyses. Following the rating of network success, a qualitative study will be conducted to provide a more in-depth examination of the most successful networks.

**Discussion:**

This is the first study to combine quantitative and qualitative methods to examine the factors that contribute to the success of clinical networks and, more generally, is the largest study of clinical networks undertaken. The adaptation of expert panel methods to rate the impacts of networks is the methodological innovation of this study. The proposed project will identify the conditions that should be established or encouraged by agencies developing clinical networks and will be of immediate use in forming strategies and programs to maximise the effectiveness of such networks.

## Background

### The role of clinical networks in improving evidence-based practice

It is widely accepted that patients who receive evidence-based care achieve better outcomes. However, despite increases in more rigorous clinically relevant research, the slow and haphazard uptake or failure to adopt such evidence into practice persists [1,2].

Clinical networks are more commonly being viewed as an important strategy for increasing evidence-based practice and improving models of care [3]. It is argued that clinical networks provide 'bottom up' views on the best ways to tackle complex healthcare problems and can facilitate or champion changes in practice at the clinical interface [3,4]. Most clinical networks are established to improve the quality of and access to care for patients, including those who require care across a range of care settings. The term *clinical network *has been used to describe many variants of networks, ranging from fully integrated service delivery systems to informal communities of practice [3]. In this study, we define the term clinical networks to mean voluntary clinician groupings that aim to improve clinical care and service delivery using a collegial approach to identify and implement a range of strategies.

### Clinical networks in New South Wales, Australia--focus of the study

An example of such an approach in Australia is the New South Wales (NSW) Agency for Clinical Innovation's (the Agency) non-mandatory model of clinical networks. The Australian health system is a mix of public and private providers. The federal government is responsible for national initiatives and policies, regulation, and funding, while the state governments are responsible for the delivery and management of hospital services. The Agency is a board-governed statutory organisation funded by the NSW State Health Department that has been fully operational since 2004, serving as a mechanism for bringing about clinical change and improved health outcomes. These networks vary in clinical focus (*e.g*., stroke care, ophthalmology, and urology), size (43-708 members), and length of operation (14-113 months) [5]. Each network is chaired by clinicians, has a Network Manager employed by the Agency, and implements its chosen activities in association with the State Health Department and the relevant area health service/s. (Further operational details are provided in Table 1 and in a recent Sax Institute report [5].)

**Table 1:** The New South Wales Agency for Clinical Innovation clinical network model

Network Feature	Description of Network Model
**Goal**	The goals of the networks are to improve health services and health outcomes by developing services based on clinical need, improving the quality of care and safety for patients, increasing equity of access and equity of outcomes within the hospital system, and enabling clinician-and consumer-driven planning.

**Membership**	The clinical networks are composed of volunteer health professionals across a range of clinical areas to disseminate knowledge on evidence-based care.

**Structure**	The networks are free to select those issues that they believe will be effective in improving care. Each network is chaired by clinicians, has a Network Manager employed by the Agency, and implements activities in association with State Health Department and the Area Health Services.

**Inputs to support networks**	To support the networks, the Agency provides • funds to employ a Network Manager and approximately AUD$30000 for small projects and operations; • training and support for the Network Managers; • funds for larger-scale projects on a competitive basis of approximately AUD$100000 (per project); • accommodation and office facilities for the Network Managers, most of whom are located together in the main Agency office; • bimonthly meetings for Network Managers to report on activities, discuss common problems, and share ideas and potential solutions; • monitoring of progress and feedback by close involvement in network activities, ongoing supervision of managers, and overview of annual reports from each network that address their activities against their annual plans; • profiling of the work of the networks through formal annual reports.

### The evidence gap: What makes clinical networks successful?

Some clinical networks are more effective than others. Clinical networks can engage clinicians in service redesign and reform [6,7], develop and implement protocols [6,7], develop and implement guidelines [8-10], facilitate knowledge sharing [9], and design and implement quality-improvement programs that result in improved quality of care in hospitals [7,8,10]. However, other research has reported that clinical networks have not had an impact [6]. In studies evaluating more than one network, varying success between networks has been reported [11], with others being unable to sustain improvements after the funding cycle ended [12].

Much of the research into clinical networks focuses on describing the establishment and activities of single networks [3]. Few studies have aimed to identify critical factors that determine the effectiveness of a network [11]. A recent Swedish qualitative study compared factors associated with three successful clinical networks with three networks that did not develop successfully [13]. Three major determinants of developing a successful network were identified: professional dedication, legitimacy, and confidence. However, this study examined only a small number of networks, provided limited information regarding study design and methods, and did not quantify the strength of any observed association.

Given their widespread implementation and data indicating variable success, there is considerable interest in understanding how clinical networks can best be established and supported to maximise their impact on patient care and service delivery.

### Aim

The study takes advantage of a unique opportunity provided by the Agency's non-mandatory model of clinical networks to investigate the factors that contribute to the success of clinical networks. Multiple coexisting networks, such as those operating under the Agency, provide an opportunity to holistically examine the range of factors that affect the success of clinical networks.

### Research objective and hypotheses

The objective of this study is to investigate the external support, organisational, and program factors associated with successful clinical networks. Based on our conceptual model described below, success is defined as follows:

**• Healthcare impact: **The extent to which there is evidence of impact on healthcare and patient outcomes.

**• System impact: **The extent to which there is evidence of impact on system-wide change.

**• Programs: **The number of quality-improvement initiatives undertaken and the quality of their design.

**• Engagement: **The extent of engagement by network members in network activities.

**• User perception: **The extent to which stakeholders perceive the networks as effective and valuable.

**• Financial leverage: **The value of any additional resources leveraged.

We hypothesise that clinical networks will be more successful if they have these features, based on Paul Bates and colleagues' theory of change in healthcare [14]:

(i) A high level of **external support **from area health service and hospital management.

(ii) Effective **organisation**, specifically strong clinical leadership and efficient internal management.

(iii) **Well-designed quality-improvement programs**, specifically those that are based on an analysis of the problem, have a specific targeted structural or behavioural change, have an explicit implementation plan, and monitor impact.

### Conceptual model

Given the heterogeneity of clinical disciplines and health conditions focused upon by clinical networks, multiple metrics of the success of networks is required [15]. For example, disease-free survival, readmission rates, or mortality rates will vary in applicability for different networks. A key component of our approach was to develop a defensible suite of outcomes to judge the successfulness of clinical networks that are justifiable to scientific, clinical, and policy communities.

**Figure 1:**
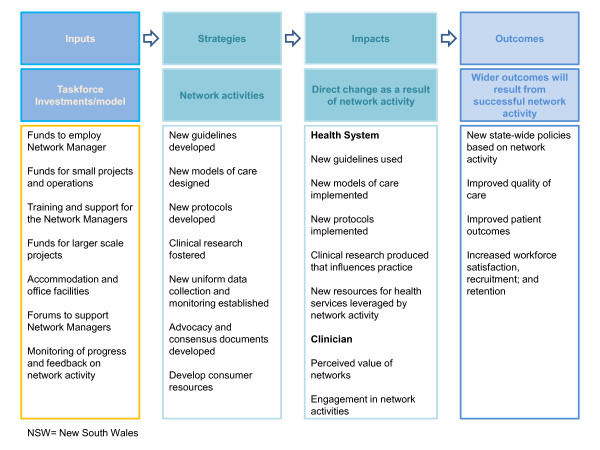
**NSW Agency for Clinical Innovation - Clinical Networks Project Logic Framework**.

In partnership with the Agency board, executive, and staff, the research team iteratively developed the program logic framework that underpins the model of the Agency's networks. Figure 1 shows the ways in which the actions of the networks are anticipated to improve healthcare and health outcomes. This logic, the outcomes of successful networks, and explanatory factors were further explored in a qualitative study with 27 stakeholders [16] of the clinical networks to inform the overarching conceptual model for this study and the subsequent design of data collection methods.

**Figure 2:**
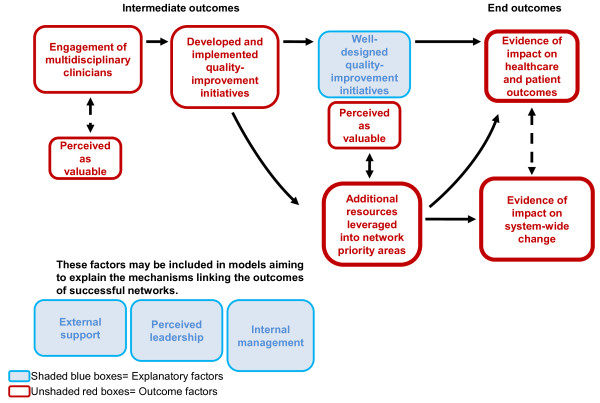
**Representation of a causal pathway for the outcomes of effective networks**.

Figure 2 presents a conceptual model linking our hypothesised outcomes and explanatory factors. The face validity of this model was confirmed with board and executive members of the Agency as well as the managers of the networks.

The outcomes have potential to influence each other, and in many ways, could be interdependent. For this model, the outcomes have been grouped into 'end outcomes', which are more long-term indicators of success, and 'intermediate outcomes', which may function as indicators of success independently or as intermediary steps towards success illustrated in another way. The explanatory factors underneath the model could be relevant at different stages along this pathway, potentially having an influence on the different outcomes. These factors could function to 'enable' the outcomes and, as such, could be included in the model contributing to any of the outcomes.

## Methods

### Design

**Figure 3:**
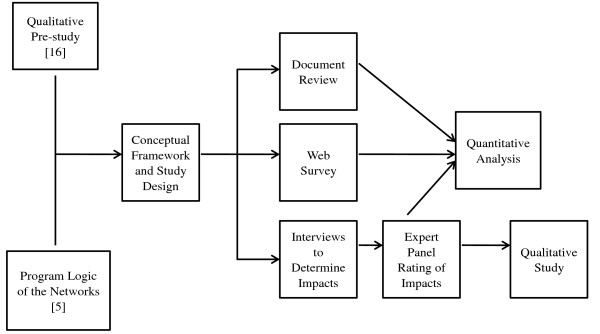
**Study overview**.

This paper describes the protocol for a retrospective study of the association between external support, organisational, and program factors (explanatory factors) and indicators of success (outcomes) among 19 clinical networks over a three-year period (2006-2008). The unit of analysis for this study is the network (see Table 1 for more operational details of the model of clinical networks). This study will examine initiatives undertaken over a three-year period between 2006 and 2008. We have selected a three-year intervention period to balance the time required for evidence of impact against accuracy of recall. At the commencement of the proposed study in 2010, all of the networks had been in operation for longer than three years and most for longer than four years. Due to the complexity of the study, it will include a series of approaches (see Figure 3 for a study overview). Firstly, information about the outcomes and exposures will be collected using a web-based survey, interviews, and record review. Based on these data, an independent expert panel will provide judgements about the impact on health and system outcomes. Panel ratings will be the outcome used in statistical analyses.

Following the rating of network success, a qualitative study will be conducted, complementing the quantitative study. This will assist with interpretation of the results by providing more in-depth examination of factors that contributed to the successful networks. We will purposely select up to three networks to focus on in more detail. In-depth interviews with key informants associated with those networks will explore the reasons for the associations we may find between explanatory factors and outcomes.

### Outcome indicators (see Additional file 1)

**• Evidence of impact on healthcare and patient outcomes: **This study requires a standard approach to measure changes in quality of care and patient outcomes, taking into consideration that the networks have developed different initiatives focused on a wide range of different conditions dealt with by different health services. Using the definition in Additional file 1 secondary evidence of each network's impact on healthcare and patient outcomes will be collected through interviews with network leaders and managers. This evidence will then be submitted to the expert panel for rating on the extent of impact on quality of care and patient outcomes for each network.

**• Evidence of impact on system-wide change: **The Institute of Healthcare Innovation in the United States has advocated for the wider adoption of network initiatives throughout the health system as a measure of network success [17-19], and this has been used to assess health system performance [20,21]. The secondary evidence of each network's impact on system-wide change will be collected through interviews with Network Chairs and Managers. An expert panel will rate the extent of impact on system-wide change for each network based on this secondary evidence.

**• Developed and implemented quality-improvement initiatives: **A census of network activities will be identified through a review of minutes of network meetings, annual plans, and other relevant existing documents. Details of quality-improvement initiatives will be corroborated through interview and by sighting secondary supporting evidence [22].

**• Engagement of multidisciplinary clinicians: **Describing and classifying members of clinical networks has often been used to judge the success of clinical networks [6,13,23], along with tracking attendances, membership [11,24], and perceptions of engagement [25] through in-depth interviews and focus groups [6,9,13,26,27]. We will assess multiple dimensions of engagement using both record review and surveys to assess the extent and depth of engagement of network members.

**• Perceived as valuable: **Previous studies of clinical networks have assessed perceptions of the value and effectiveness of networks using semi-structured interviews [6,11] and focus groups [9] with patients, health service personnel, and clinicians. It is not possible to use these existing questions verbatim because they are focused on specific networks, but we will use these as a guide and draw upon key words and themes from the qualitative study [16] to design the survey to assess perceived value.

**• Leveraged additional resources: **Resources obtained for network activities from other sources apart from the Agency will be extracted from financial records using audit methods [28].

### Explanatory factor indicators (see Additional file 2)

**• External support: **Clinical networks operate within a complex political, cultural, and organisational context [13]. Although Turrini and colleagues [29] identified community cohesion, local support, and participation as critical factors in the success of networks, few studies have considered the external context in which networks operate [12,13]. We developed questions for our web survey based upon keywords and themes from the qualitative study [16], which strongly supported assessing relationships with health agencies as a determinant of network success. Perception of network members about aspects of external context defined in Additional file 2 will be assessed through the web survey.

**• Perceived leadership: **A growing body of research evidence supports the influence of leadership on the success of networks [6,9,22,30]. Using a web survey of network members, we will assess the strength and quality of the leadership of the network across six key aspects derived from previous literature (see Additional file 2).

**• Internal management: **Models of effective healthcare organisations emphasise the importance of efficient internal management [14,31] and the impact of internal structures (*e.g*., size, staffing, governance) and processes for facilitating communication and knowledge sharing between network members [9,11,32]. Previous studies of clinical networks have predominantly used document review and semi-structured interviews to assess internal management [9,32,33]. Aspects of internal management of each network (defined in Additional file 2) will be assessed through record review and perceptions of network members in the web survey.

**• Well-designed quality-improvement initiatives: **Each network will be categorised on how well the quality-improvement initiatives that contributed to their main outcomes were designed in terms of the four criteria detailed in Additional file 2[34].

### Data collection methods and samples

**• Web survey: **The aim of the web survey is to assess network members' perceptions of value, effectiveness, leadership, management, external support, and engagement. The web survey has been developed by building upon appropriate existing measures relating to clinical networks and those in the wider organisational literature (see Additional file 2 and above). In addition, all questions have been tailored to the local context by taking account of the views and vocabulary elicited in qualitative exploratory interviews with key stakeholders who have explicit knowledge of the networks of the Agency [16]. A record review (Agency minutes and membership lists) was used to identify a total of 4,280 individuals who participated in the 19 clinical networks of the Agency from January 2006 to December 2008. All 3,316 network members and participants with known email addresses will be contacted and invited to participate in the web survey. The web survey will ask retrospective questions about the attitudes and perceptions of network members and participants during the study period (2006-2008). To aid recall and minimize recall bias, a number of measures will be employed, including [35,36] (a) recall prompts to assist respondents to identify the relevant time period and (b) recall aids to enable respondents to use recognition rather than recall as a strategy for reporting specific activities/quality-improvement initiatives since these questions may be easier to answer if referring to specific initiatives.

**• Interviews: **19 interviews to determine the impacts of the networks will be conducted with both managers and clinical chairs at the same time for each network. The aim of this interview is to identify the most important impacts on quality of care and system-wide change that resulted from their network's activities between 2006 and 2008. Participants will be asked (a) to identify the most important impacts, (b) to explain the importance of each impact, (c) how their network activities led to that impact, and (d) for evidence of the impacts. The interview will not be a qualitative exploration but rather an evidence-gathering activity. The interview will also be recorded as a back-up (not transcribed). The Network Managers will also be required to obtain evidence to demonstrate the impacts and how those impacts relate to the network activities. Following this interview and receipt of the evidence, a *pro forma *detailing the four areas covered in the interview listed above for each impact will be completed by the study team and reviewed by Network Managers and Chairs for accuracy. These *pro formas *and associated evidence will be passed on to the expert panel for rating.

**• Expert panel: **This study will use an adaptation of the RAND/UCLA (University of California, Los Angeles) appropriateness method [37,38]. This is a systematic consensus method that has been widely used to derive expert consensus on clinical indications. The traditional use combines expert opinion with a systematic review of the scientific evidence to determine whether a given procedure would be appropriate in specific situations. More recently, the RAND/UCLA approach has been adapted to assess the appropriateness of quality-improvement initiatives and whether they would be likely to improve health outcomes or healthcare quality [39-41]. Lisa Rubenstein and colleagues used the method to establish organisational quality-improvement priorities, focusing on system-based objectives rather than specific issues within patient care [40]. The panel will have a chair and four members with extensive expertise in system-wide clinical care and quality-improvement programs as well as the expert panel method. The panellists were selected through a voting and nomination process with the study investigators. In order to ensure independence of the panel members from the Agency, the study team members from the Agency were not involved in the voting and selection of panellists, and all panellists completed a conflict of interest declaration. Based on the evidence provided by the networks on quality of care and system-wide change, the panellists will individually rate the importance of each impact on a nine-point scale. In a moderated meeting, the panel will then discuss any discrepancies in their scores and rerate each impact for a final score of overall success.

**• Document review: **The Agency has kept detailed records of all meetings and initiatives and provides regular reports to the NSW Department of Health and to the Minister. These records will be audited using a standardised coding schedule, including free-text annotations, to identify initiatives undertaken and network membership. Additional resources leveraged will be extracted from income and expenditure statements in financial records.

### Data analysis

#### Statistical methods--association between outcomes and exposures

The unit of analysis for this study is the network. Nonparametric Spearman correlation coefficients will be obtained to investigate the association between the various outcomes (to determine if those who score highly on one outcome also score highly on other outcomes), between the various explanatory variables, and between each outcome and each explanatory variable. Other potential factors that may confound the association between the hypothesised explanatory factors and outcomes of clinical networks will be examined. These include months of operation, Network Manager's average full-time equivalent working hours during the study period, turnover of staff, turnover of chairs, budget allocated to network by Agency, and start-up network budget. Multiple linear regression analysis will be undertaken to examine the relationship between all exposures and each of the outcomes. Variables will be included in the regression model if they have a *p *value of 0.25 or less on univariate analysis, and a stepwise process will be used to include/exclude variables until the final model is determined. Because the number of observations for these models is small (19), it will not be possible to include a large number of variables in each model. Therefore, various models will be generated for each outcome that consider meaningful groups of explanatory variables at a time. A significance level of 5% will be used to assess statistical significance in the final model. If appropriate (*i.e*., there is variation in precision of the summary measure estimates), we will use the method of Kulathinal [42] to adjust for variation in the measurement errors among networks.

### Sample size

There have been no previous quantitative studies examining the association between organisational and contextual factors and the effectiveness of clinical networks. We have used data from a recent Australian study examining the association between clinical performance and organisational determinants in 19 healthcare organisations to estimate the likely effect size; in this study, Spearman correlation coefficients for associations of relevance to our study generally range from 0.45 to 0.71. With 19 networks and a 5% significance level, we have 80% power to detect a correlation coefficient as being statistically significant if it is 0.6 or more. Thus, we will have sufficient power to detect moderate to large associations that, given the findings of Braithwaite [41], are achievable and clinically meaningful.

### Qualitative study

The final stage of this study will be a qualitative study to complement the quantitative results by exploring, from the viewpoints of key stakeholders, the functions and relationships between the network features and processes associated with making an impact on quality of care and system-wide change. The aim of this study is to develop in-depth reasons and explanations for network success that could then be used to inform future network development. The successful impacts within the networks will be explored, with the aim of gaining insight into the process(es) that led to the impact. The results of the analysis will inform (a) the selection of networks and (b) the explanatory factors that will be explored qualitatively. The data will be collected through individual, face-to-face semi-structured interviews with key informants involved in the success of each network. A snowballing approach to sampling will be used to locate the key informants, starting with a Network Chair and the Network Manager from 2006 to 2008. The sample will include a mix of those connected with the network and those who are not linked to the network to gain multiple perspectives. The qualitative exploration will involve thematic analysis of data to identify the main themes that emerge across the accounts of success as having made an impact. This study will function to illuminate aspects of the quantitative analysis and to drill down to identify why and how those features identified in the quantitative work contributed to network success.

## Discussion

This project will use the unique opportunity provided by the clinical networks of the NSW Agency of Clinical Innovation to undertake the first quantitative study to examine the factors that contribute to the success of clinical networks and, more generally, the largest study of clinical networks undertaken internationally. The mixed-methods approach combined with the adaptation of expert panel methods to rate impacts of networks is the methodological innovation of this study.

Challenges inherent in this study relate to difficulties in comparing these very different networks--the 'apples and oranges' problem. We need to rate each network's impact on quality of care and system-wide change, taking into account heterogeneity of impacts. A further challenge in this comparative study will be whether it is possible to adequately take account of other large differences between the networks that may influence the impacts they can achieve, such as the focus and size of their clinical discipline and their stage of operational establishment. Our methods, as outlined in this protocol, will go some way to addressing these challenges, but further validation is likely to be required.

The project is based on a strong working partnership between the research group and the clinical networks. This enables the research to be framed around the real-world operational issues of the networks and for the study to be designed so it is sensitive to the operational constraints of the networks. The research team has expertise in social and behavioural science, economics, clinical epidemiology, biostatistics, clinical care, and evaluation of health service interventions. Furthermore, a number of members are leaders in the implementation of clinical networks (including the CEO, Executive Director, and former Chair of the Agency). With this combination of collaborators, the study will meet scientific standards and will also be used by the Agency when setting policy directions for the networks.

There is an urgent need to understand the factors that increase the likelihood that clinical networks will be effective because they are being widely implemented in Australia and other countries. The proposed project will identify the conditions that should be established or encouraged by agencies developing clinical networks and will be of immediate use in forming strategies and programs to maximise the effectiveness of such networks. The findings will form the basis of strategies to improve less effective networks and to ensure that any new networks are established as well as possible. The outcomes and tools developed as part of this project can be adopted by this Agency and others for ongoing monitoring of impact.

## Competing interests

Hunter Watt and Kate Needham, who are part of the 'Clinical Networks Research Group', are employed by the NSW Agency for Clinical Innovation. This Agency has provided funds to support this research as part of the National Health and Medical Research Council of Australia's (NHMRC) partnership project grant scheme. These funds have been awarded on the basis of a NHMRC deed of agreement governing the governance and conduct of research in Australia. The other authors declare that they have no competing interests.

## Authors' contributions

The authors are the chief, associate, and honorary investigators of the research grant funding this research activity. MH, in collaboration with all other authors, conceptualised the research project and developed the protocol presented in this paper. All authors provided input into various aspects of the study, provided ongoing critique, and approved the final version of the manuscript.

## Supplementary Material

Additional file 1 **Summary of outcomes, indicators and data collection method of successful networks**. Click here for file

Additional file 2 **Summary of explanatory factors, indicators and data collection method of successful networks**. Click here for file
