# Supplementary material for: Determinants of successful clinical networks: the conceptual framework and study protocol
Source: Implement Sci. 2012 Mar 13;7:16. doi: 10.1186/1748-5908-7-16 (PMC3328243; doi:10.1186/1748-5908-7-16)
Supplement: Additional file 1 — Summary of outcomes, indicators and data collection method of successful networks. [file 1748-5908-7-16-S1.DOC]

**Additional file 1: Summary of outcomes, indicators, and data collection methods of successful networks**

| **Variable** | **Definition** | **Method** | **Pilot Indicators** |
| --- | --- | --- | --- |
| Evidence of impact on quality of healthcare | Evidence that networks have made an improvement to the safety, effectiveness, appropriateness, accessibility, efficiency, and patient-centred nature of care. Where possible, such improvements will be illustrated with direct evidence of changes in practice or using patient-level data to illustrate a change. Impacts may relate to a single network activity or a combination of a number of network activities. These impacts need to have arisen from activities conducted between 2006 and 2008. | Rating of impact by expert panel  (evidence provided through interviews) | Median score from expert panel  All panellists will give scores between 1 and 9 for the extent of impact.  (The rating will be given after reviewing evidence supplied by networks following an interview with Network Executives, Chairs, and Managers.) |
| Evidence of impact on system-wide change | The adoption of network initiatives on a larger scale, making improvements to the wider health system. Such implementation of changes could be on a moderate scale or across an entire system and could vary with the innovation being scaled up, the issue it addressed, and the context in which the change was being initiated. Impacts may relate to a single network activity or a combination of a number of network activities. These impacts need to have arisen from activities conducted between 2006 and 2008. | Rating of impact by expert panel  (evidence provided through interviews) | Median score from expert panel  All panellists will give scores between 1 and 9 for the extent of impact.  (The rating will be given after reviewing evidence supplied by networks following an interview with Network Executives, Chairs, and Managers.) |
| Developed and implemented quality-improvement initiatives | Creating and planning a range of activities to improve the provision of evidenced-based care. These initiatives will have been fully developed; however, they may not have been implemented. | Record review of network meeting minutes verified with interviews and viewing of secondary documents & communication with Network Managers. | Number of activities completed by each network.  For descriptive purposes, these are categorised into models of care, clinical guidelines, care protocols, policy, system or processes, education for implementation into practice, advocacy, data collection monitoring systems, workforce improvement initiatives, consumer resources, clinical research and scholarship, and quality-improvement evaluation. |
| Engagement of clinicians | Engaging clinicians in networks is a fundamental part of their existence.  A strong theme that emerged from a qualitative study [14] conducted with NSW stakeholders of networks was that of connecting and engaging clinicians in networks in terms of:   - Interdisciplinary collaboration - Collaboration with consumers - Engagement with rural/remote sector | Record review of network meeting minutes and ACI records of network members  Web survey | From document review:   - Number of members/ participants per network (document review) - Number of senior medical and nursing members (document review) - Number of medical, nursing, and allied health and consumers per network (document review) - Number of members and participants in rural and regional Australia (document review) - Average score on questions of depth of engagement by people linked to each network (Likert scale): - Number of hours devoted to network activities in last 6 months (estimate of time) - Perceived commitment to network and belief in the work it undertakes - Perceived involvement in day-to-day work of the network - Input perceived as less visible behind the scenes - Input perceived as driving the network agenda |
| Perceived as valuable | Perceptions about the worth of the ACI clinical networks, including the belief that the networks have the scope to make a contribution to health service provision in NSW. | Web survey | A range of response options on questions of perceived value by people linked to each network :   - Perception that the network has improved quality of care - Perception that the network has improved patient outcomes - Perception that the network has led to system improvements - Likelihood of recommending joining this clinical network to a colleague - Perception that network involvement helps professionally |
| Leveraged additional resources | The ability to leverage additional resources into network priority areas over and above direct ACI funding for operating costs and special projects, indicative of support from outside agencies and of the perceived value of network initiatives. | Record review of financial reports from each network | Additional resources obtained for ACI activities from other sources apart from ACI   - Australian dollars per network |
| NSW = New South Wales; ACI = NSW Agency for Clinical Innovation. | | | |
