# Supplementary material for: Determinants of successful clinical networks: the conceptual framework and study protocol
Source: Implement Sci. 2012 Mar 13;7:16. doi: 10.1186/1748-5908-7-16 (PMC3328243; doi:10.1186/1748-5908-7-16)
Supplement: Additional file 2 — Summary of explanatory factors, indicators and data collection method of successful networks. [file 1748-5908-7-16-S2.DOC]

**Additional file 2: Summary of explanatory factors, indicators and data collection method of successful networks**

| **Variable** | **Definition** | **Method** | **Pilot Indicators** |
| --- | --- | --- | --- |
| External support | The alignment of network agendas and strength of relationships with external agencies, including NSW Health, AHS, and hospital management. Specifically:   - Network workplans and agendas aligned with state government strategic plans - Strong relationship with NSW Health - Strong relationship with AHS Managers - Support from hospital management | Web survey | Average score on questions on the perceived external context by people linked to each network (Likert scale):   - Support from hospital management - Strong relationship with AHS Managers - Strong relationship with NSW Health - Network workplans and agendas aligned with state government strategic plans |
| Perceived leadership | Strength and quality of the transformational and transactional leadership of the network, including Network Managers, clinical chairs, and the ACI Executive across six key aspects:  Transformational leadership:   - Vision and facilitation - Motivation - Building collaborative relationships and engaging with the external environment - Role model   Transactional leadership:   - Clearly defined goals and achievable work plans - Ability to implement change | Web survey | Average score on questions of perceived leadership by people linked to each network will be scaled up into an overall score for the Network Manager, Clinical Chair, and ACI Executive for:   1. Transformational leadership 2. Transactional leadership |
| Internal management | The efficiency of the internal management of the networks across the following dimensions:   - Composition of the network steering committee (number of clinicians, consumers, stakeholders) - Frequency of network meetings - Open and facilitative approach to management - Working groups convened to progress projects - Clearly stated written governance and decision-making processes (*e.g.*, a strategic plan, mechanism for resolving conflict) - Formalised communication and dissemination strategies (*e.g.*, education, training, knowledge sharing, and learning activities) | Document review of network meeting minutes and memberships  Web survey | From document review:   - Number/proportion of multidisciplinary members of executive committees - Number/proportion of committee members who are heads of discipline within their hospital - Number of meetings over period - Number of working groups who meet and complete a project - Number of strategic plans per annum   Average score on questions on the perceived efficiency of internal management by people linked to each network (Likert scale):   - Perceived multidisciplinary representation - Perceived dominance of individuals - Perceived supportiveness - Perceived effectiveness of information sharing across the network - Perceived effectiveness of communication with people outside the network - Perceived organisational ability of the Network Manager |
| Well-designed quality-improvement initiatives | One element of network success may relate to how well their quality-improvement initiatives were designed and implemented.  *Quality-improvement initiatives* have been defined by Lisa Rubenstein and her colleagues as those initiatives that ‘seek to improve the quality of care delivered by target organisations or organisational units such as practices, intensive care units or community groups’, addressing ‘both the method(s) for encouraging change and the intended changes themselves’ [40]. | A rating by the research team on how well the quality-improvement initiatives that contributed to their main outcomes were designed. | The four criteria [34] were derived and have been defined as follows:   1. Clear expression of what the quality-improvement initiative was aiming to change 2. A conceptual model and evidence to support why the initiative might be expected to work 3. Articulated plans for implementation—who it might involve, how it was communicated or implemented, whether it was appropriate to be generalised in ‘natural settings’, and the timescale of the initiative 4. Ongoing monitoring of how the quality-improvement initiative was being implemented and evaluated |

NSW = New South Wales; AHS = Area Health Services; ACI = NSW Agency for Clinical Innovation.
